# Characteristics of Plant Eating in Domestic Cats

**DOI:** 10.3390/ani11071853

**Published:** 2021-06-22

**Authors:** Benjamin L. Hart, Lynette A. Hart, Abigail P. Thigpen, Neil H. Willits

**Affiliations:** 1 Department of Anatomy, Physiology and Cell Biology, School of Veterinary Medicine, University of California, Davis, CA 95616, USA; 2 Department of Population Health and Reproduction, School of Veterinary Medicine, University of California, Davis, CA 95616, USA; lahart@ucdavis.edu (L.A.H.); apthigpen@ucdavis.edu (A.P.T.); 3 Department of Statistics, University of California, Davis, CA 95616, USA; nhwillits@ucdavis.edu

**Keywords:** felids, grass eating, hairballs, long-haired cats, vomiting

## Abstract

**Simple Summary:**

Plant eating by domestic cats is of interest to veterinarians and cat owners. Two surveys of cat owners were launched testing important questions regarding whether plant eating: (1) is a response to the cat feeling ill; (2) induces vomiting; (3) is a means of expelling hair balls from consumed hair. Based on observations of wild felids, it was also considered that plant eating may reflect an innate predisposition acquired from the ancestral cat. This study found that very few cats showed signs of illness before eating plants. However, 27 to 37 percent of cats, respectively in the two surveys, frequently vomited after eating plants; this indicates that gastrointestinal disturbance may be related to vomiting in some cats. Young cats consumed plants more frequently than older cats and young cats also appeared ill and vomited less frequently than older cats in association with plant eating. Short-haired cats ate plants as frequently as long-haired cats, suggesting that plant eating is not for expelling hairballs. Some guidelines for cat owners with indoor cats are provided.

**Abstract:**

Plant eating by domestic cats is of interest to veterinarians and cat owners, especially with the current trend to keep cats totally indoors. Feline grass gardens are commonly provided to such cats as a reflection of cat owners believing in the need or desire of cats for eating plants. Two surveys with 1000 to 2000 returns from cat owners were launched over 10 years to test different hypotheses regarding plant eating. These hypotheses are that plant eating: (1) is a response to the cat feeling ill; (2) induces vomiting; (3) is a means of expelling hair balls from consumed hair. Additionally, a perspective acquired from observations of wild felids is that plant eating reflects an innate predisposition acquired from the ancestral cat. In this study, very few cats showed signs of illness before eating plants. However, 27 to 37 percent of cats, respectively in the two surveys, frequently vomited after eating plants, indicating that gastrointestinal disturbance may be related to vomiting in some cats. Young cats consumed plants more frequently than older cats and appeared ill and vomited less frequently in association with plant eating. Short-haired cats ate plants as frequently as long-haired cats, arguing against the hairball expelling hypothesis. Some guidelines for cat owners with indoor cats are provided.

## 1. Introduction

Plant eating by dogs and cats has long piqued human curiosity. In an ancient explanation of this behavior, Aristotle claimed that dogs never eat grass, except when they are sick to bring on vomiting and purge themselves [1,2]. In current times, many caregivers frequently see their domestic cats and dogs eating grass and other non-digestible plants and may wonder about the reason for this behavior. The behavior has been studied in dogs, where it was found that 68 percent ate plants on a daily or weekly basis [3]. Nine percent were reported to appear ill frequently before eating plants and 22 percent to vomit frequently afterwards. Dogs of a younger age ate plants more frequently than older dogs. Due to the lack of a clear relationship of feeling ill or vomiting and plant eating in that study, it was concluded that plant eating was an innate predisposition of dogs. 

Based on the findings regarding plant eating in dogs, the goal of the present study was to characterize cats’ behavior and frequency of plant eating, and to test the common hypotheses regarding them appearing ill before eating plants, vomiting afterwards, and expelling hairballs. The latter hypothesis is based on the observations that long-haired cats consume a greater quantity of hair than short-haired cats, and that they more commonly vomit hairballs (trichobezoars) [4]. 

Understanding plant eating of household cats is particularly challenging because many companion cats are kept indoors with no exposure to edible plants. Others live as indoor-outdoor cats where they are not regularly observed with regard to plant eating. Therefore, the questions addressed with some testable hypotheses were explored with cat owners who could regularly see their cats and would notice plant eating. Lifestyle factors and demographics of the cats, such as the source, age, sex, and neuter status of the cat, number of cats in the household, and indoor only versus indoor-outdoor maintenance were also examined. For this study, we formulated three testable hypotheses with the intention of reaching conclusions regarding the best explanation for cats’ plant-eating behavior. Another explanation for feline plant eating, addressed in the Discussion, is that plant eating reflects an instinct inherited from wild ancestors for periodic purging of intestinal parasites. This explanation could not be tested as a hypothesis, but it would gain weight if the testable hypotheses are not strongly supported. The following are the testable hypotheses:

1. Most cats that engage in plant eating will exhibit signs of illness prior to eating plants, a behavior that can be noticed by the caregivers who are familiar with their behavior.
2. Most bouts of plant eating induce vomiting within a few minutes after the plant eating.
3. Plant eating facilitates purging of ingested hair and occurs more frequently in long-haired cats than short-haired cats. This was tested by comparing cats identified as being domestic short hair versus domestic long hair, and Siamese (short-haired) versus Burmese (long-haired).

These hypotheses were addressed in two surveys administered to cat owners. The first two hypotheses were assessed by determining the proportion of responses reporting cats showing signs of illness before eating plants or vomiting afterwards, using the rule of thumb that less than 30 percent overall indicates little support and more than 30 percent indicates at least partial support for the hypothesis. The third hypothesis was assessed by comparing long-haired with short-haired cats for statistically verified differences. 

## 2. Materials and Methods

This study included two surveys of cat owners. Survey 1, a web-based survey, was launched in 2006, entitled, “Cats and plants: A scientific study of grass- and plant-eating behavior in cats.” The survey was intended to recruit anonymous participants specifically to answer questions about plant eating (Survey Suite, University of VA, Charlottesville, VA, USA) and was designed for internet distribution, such that it would take approximately 20 min to complete. The questions were developed and edited by the authors (BLH, APT) after giving preliminary copies to clients of the veterinary hospital. Similar web-based surveys have been used in a variety of data-based behavioral and medical investigations, such as for evaluation of asthma treatments [5], practice patterns in treatment of urinary incontinence [6], and plant eating in dogs [3]. Studies have shown that the quality of the data from web-based surveys is comparable to traditional survey methods [7]. The survey is available as Supplementary File S1 and in Figshare.

Survey 2, also web-based, was launched in 2016, and was intended to collect general information on the demographics of plant eating, types of plants eaten, and effects of age, sex, and gonadal hormone status of the cats. Since breed identification was included, long-haired cats could be compared with short-haired cats. The survey was labeled: “Life with Your Cat: Understanding Some Feline Behaviors”, so as to avoid recruiting cat owners specifically on the plant eating topic. Questions about plant eating were mixed in with general questions about various behavioral issues. Using an established web-based program, SurveyMonkey, the survey was designed for internet distribution, such that it would take approximately 20 min to complete. The survey included questions dealing with the description of the cat and its environment, behavior, and plant eating. For households with more than one cat, respondents were asked to answer the questions for the cat they had known the longest; this was the “designated cat.” The survey is available as Supplementary File S2 and in Figshare.

The returns from the surveys were filtered with two exclusion criteria: (1) the cats were outdoor, free-ranging and could not be regularly observed; and (2) the cats were strictly indoors with no access to edible plants. The inclusion criteria for indoor-outdoor and indoor cats were: (1) the respondent (cat owner) had to have known the cat for at least 11 months, (2) the respondent had to have been able to see the cat’s behavior generally three or more hours a day, and (3) indoor cats had to have access to non-toxic plants.

### Statistical Analyses

The analyses used involved comparing cats in various age groups for an increasing or decreasing trend in the frequency of the behavior in question according to the Mann–Kendall test based on the Kendall’s tau [8]. These tests were run in R using the Kendall package. The full datasets are available in Figshare.

## 3. Results

In Survey 1, 2245 responses were returned. This number was reduced by exclusion and inclusion criteria. Because of missing data for specific questions, the total number of cats varied for different considerations from 1872 to 2036.

Analyses revealed that overall, 65 percent of cats ate plants weekly. Overall, just 6 percent of cats appeared ill before eating plants. Among those that ate plants, 37 percent overall were reported as frequently vomiting afterwards. In Table 1, data are presented comparing various aspects of plant eating as a function of different age ranges. Across the age ranges, there was a significant inverse trend between age and the frequency of eating plants on a weekly basis (*p* < 0.0001). There was a significant positive trend with regard to age and appearing ill before eating plants (*p* < 0.0001), and vomiting after eating plants (*p* < 0.0001), with appearing ill maxing out at 9.4 percent for cats in the oldest age group (>9 years) and vomiting frequency maxing out at 57.1 percent for the oldest age group. The youngest cats were reported as eating non-grass plants more frequently (69.1%) than older cats (*p* < 0.0001). For cats of one year of age or older, grass was the most frequently eaten plant, whereas for cats below one year of age, non-grass plants were more commonly eaten, and grass was eaten just 39 percent of the time.

The low proportion of cats that appeared ill before eating plants does not support the hypothesis that plant eating is triggered by the cat feeling ill. The high proportion of cats a year of age or older that frequently vomited after eating plants, ranging up to 57.1 percent, suggests that in a fair proportion of cats, plant eating possibly induces vomiting. The results also show that in cats over 1 year of age that frequently ate plants, 34.4 percent (704/2048) were reported to have plants in their feces and 69.4 percent (1421/2048) to have plants in their vomit.

For Survey 2, there were 2296 returns. Applying the exclusion and inclusion criteria yielded 1021 returns. In this survey, the cats were not evaluated on the basis of age. As shown in Table 2, 71 percent of all cats were seen eating plants at least 6 times, 61 percent over 10 times, and 11 percent never seen eating plants. Of cats seen eating plants at least 10 times, 67 percent were estimated to eat plants at least weekly. When asked about their cat’s behavior prior to eating plants, 9 percent of the respondents overall said their cat frequently appeared ill. After the cat ate plants, 27 percent overall said the cat frequently vomited. 

These responses were in line with those from Survey 1, in which an overall 6 percent of cats were reported as generally appearing ill before eating plants and 37 percent were reported as vomiting afterwards. Among cats of 3 years of age or less, 39 percent engaged in daily plant eating compared to 27 percent of older cats. These data are similar to those from Survey 1, which show an inverse relationship of plant eating frequency with increasing age.

In this survey, respondents were asked about the breed of their cat, and we could compare domestic short hair cats with domestic long hair cats, and the short-haired Siamese with the long-haired Burmese—two breeds that were reported in sufficient numbers to make meaningful comparisons in plant eating behavior. Since long-haired cats reportedly have more hair balls, comparing plant eating between the two hair types would reveal if plant eating had been adopted in some cats as a means to expel hair balls. In Table 3, data from domestic short-haired cats and domestic long-haired cats show no evident differences in their degree or frequency of plant eating, nor any differences in them appearing to feel ill before eating plants or vomiting afterwards. Similarly, no differences were reported between the long-haired Burmese and the short-haired Siamese, although the purebreds were seen eating plants less frequently and reported as appearing ill more frequently before eating plants than the domestic short-haired and long-haired cats. The data do not support the hypothesis that plant eating is a behavior acquired for purging ingested hairballs or clumps of hair.

Comparing cats that were seen eating plants at least 10 times with those never seen eating plants, no differences appeared in age ranges, neuter status, sources of the cat (breeder or shelter), breeds, or numbers of other cats in the household, or whether or not they responded to catnip in either web surveys.

## 4. Discussion

This study addressed a question that cat owners and veterinarians commonly ask as to why cats eat grass or other plants. A challenge in conducting this study was the variety of different conditions in which domestic cats are maintained—some live outdoors only and are not seen consistently by the caregivers; others are indoor-outdoor cats and are seen consistently by the caregivers, and others live indoors only but have no plants available. The responses were filtered to include only responses from cat owners who could usually observe their cats for 3 or more hours a day. The issue of plant eating was addressed in two phases, in 2006 and 2016, each with a different survey to which exclusion and inclusion criteria were applied. 

Plant eating is indeed a frequent occurrence. In Survey 2, which was more demographically based, only about 11 percent of cats apparently did not eat plants. Younger cats of 1 to 2 years or 1 to 3 years of age ate plants more frequently than older cats, suggesting that the plant eating behavior is not learned from older cats.

A major goal of the surveys was to test the predictions of the hypotheses that plant eating by cats is preceded by the cats appearing ill beforehand and/or in vomiting afterwards. In Survey 1, with 1945 useable returns, 94 percent of cats appeared normal before eating plants and 37 percent were seen frequently vomiting afterwards. In Survey 2, with 1021 useable returns, the findings were similar, with 91 percent of cats appearing normal before eating plants and 27 percent vomiting afterwards. The hypothesis that cats that eat plants generally express signs of illness was not supported in these surveys. Vomiting was noticeably high enough to suggest a link to gastroenteric discomfort sometimes triggering plant eating and subsequent vomiting. 

Survey 2 was also intended to test if cats that had longer hair, and presumably ingested more hair, differed from the comparison short-haired cats. There were no evident differences between the short-haired and long-haired cats that were compared in degree or frequency of plant eating nor any differences in appearing to feel ill before eating plants or vomiting afterwards. 

The findings from this study, through the process of exclusion, support the perspective that plant eating in domestic cats, similarly to that of dogs, mostly reflects a normal, common behavior. This explanation has rich support in previous studies of wild carnivores from examination of scats and intestinal contents where it was found that both wild felids and canids regularly consume grass and other plants. In a recent extensive review of 352 studies [9], there was evidence of consumption of fibrous plant material by 124 species of carnivores. The co-occurrence of intestinal parasites with grasses or sedges in nine instances prompted the authors to propose that the fibrous plant material helps to expel the parasites. Of the studies surveyed, 81 were from the family of *Felidae* and included lynx, panthers, pumas, and leopards. There were 155 studies of *Canidae.*

Additional studies that support this perspective include observations of wolves intentionally consuming grass [10,11]. After observing blades of grass wrapped around intestinal worms in wolf scats, Murie [10] suggested that grass might have a “scouring effect” in removing worms. Another wild species studied with the intention of examining the parasite purging effect is the Chinese lesser civet. In feces collected, whole blades of grass were seen, in which adult worms of *Toxocara* sp. were trapped [12]. 

Given that virtually all wild carnivores carry an intestinal parasite load at some level, regular plant eating would have an adaptive role in maintaining a tolerable intestinal parasite load, even though the animal cannot sense the parasites. Thus, this behavior is presumably innate, and expressed in the domestic cat at some level. In nature, younger carnivores are more vulnerable to the nutritional costs of intestinal parasites, and more frequent plant eating would help maintain a lower parasite load. In this study, young cats ate plants more frequently than older ones.

A confirmatory test for the conclusion presented here would be to administer a sample of grass (e.g., in a capsule) to some cats with a calculated worm load and observe for worm expulsion compared to administering a control treatment to other cats with an identical parasite load. Due to constraints on research, such an experiment is not feasible.

These research findings also suggest that most cats have a consistent urge or compulsion to eat plants, and thus indoor cats are likely to eat or chew on house plants. Many common houseplants may be poisonous to cats, so this is an area of possible concern. The ASPCA has a website that lists safe and poisonous plants [13]. https://www.aspca.org/pet-care/animal-poison-control/toxic-and-non-toxic-plants, accessed on 21 June 2021.

**Figure 1:**
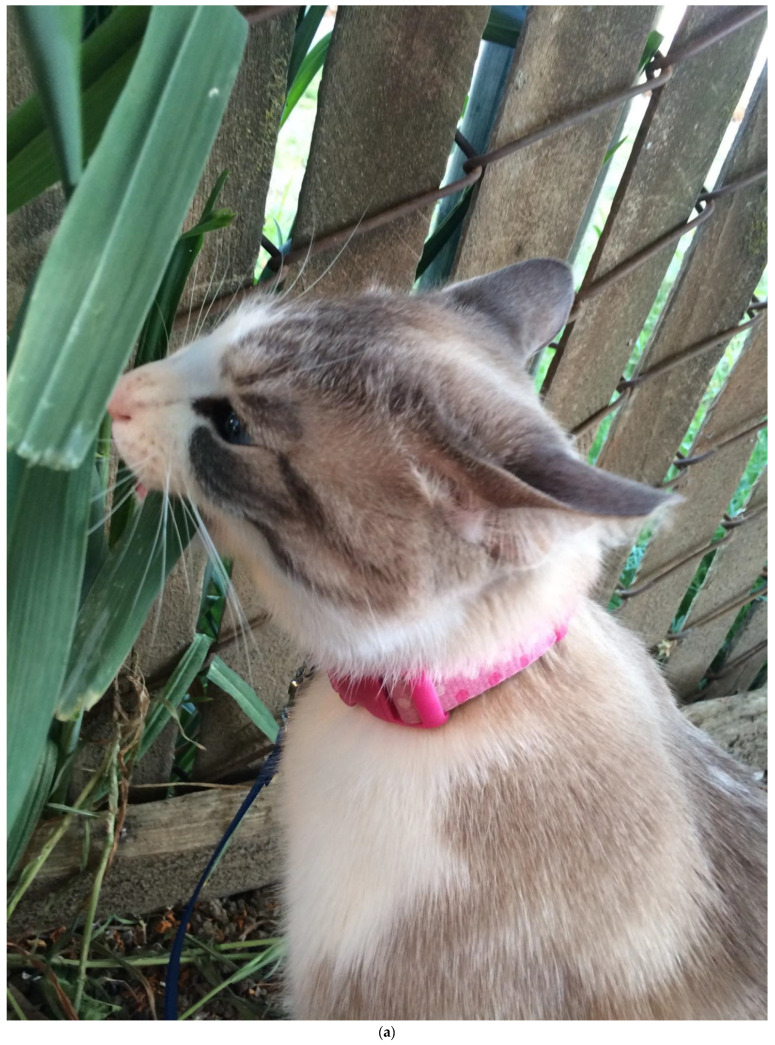

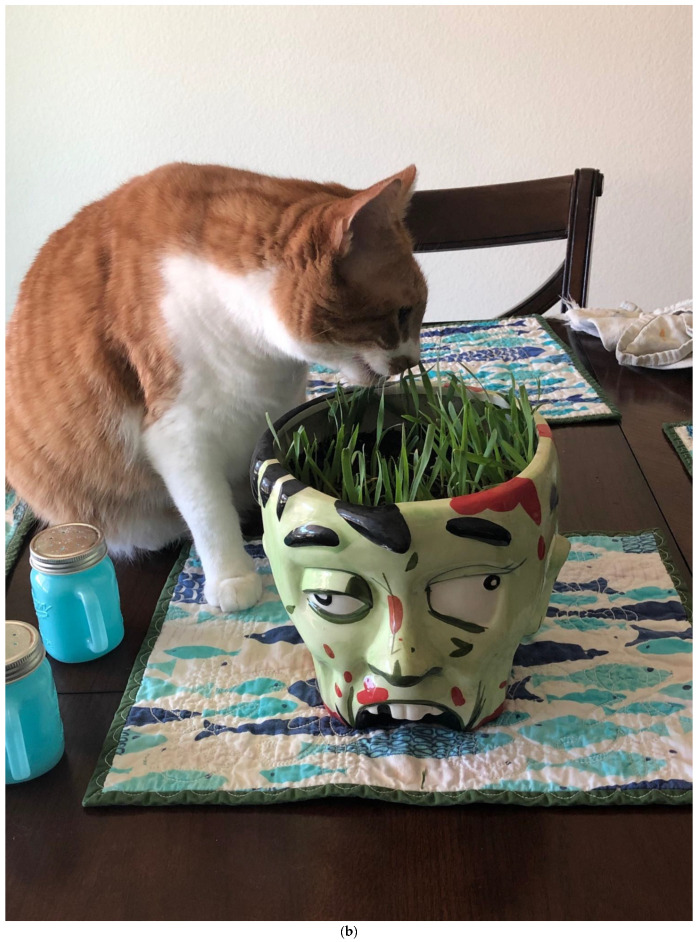
(**a**,**b**) Examples of cats eating grass inside their homes (photo credits: Erica Orcutt).

Many cat owners provide their indoor cats with a grass garden that is regularly munched on by their cats. This would appear to be a logical practice for owners of indoor cats with no outdoor access to plants. In Survey 2, 33 percent of owners of cats seen eating plants at least 10 timesprovided a bed of grass on a regular basis. Of the owners providing plants, 35 percent said that the cat “enjoyed” eating grass. Given the natural predisposition of most cats to eat plants, it seems reasonable to provide a grass garden to indoor cats who have no access to plants outdoors (Figure 1a,b).

A limitation of this study is that not all the variables that could be associated with plant eating were tested. Despite the general finding of plant eating being normal, there may be several factors that could be associated with this behavior. The possible causative factor of a gastric disturbance leading to vomiting in some cats is suggested by the results in both Surveys 1 and 2. A dietary deficiency in some trace nutrients leading to plant eating is another possibility.

## 5. Conclusions

Plant eating by domestic cats is currently seen in a variety of wild carnivores, where the behavior is linked to the control of intestinal parasites. This is a topic where the behavior of the wild relative informs the understanding of the behavior of the domestic relative. In the same process, the detailed observations of the domestic cat, such as the young animals eating plants more frequently and vomiting less frequently than older ones, suggest aspects of behavior that could be pursued in the wild relatives.

## Figures and Tables

**Table 1:** Behaviors associated with plant eating. The analyses compared cats in various age groups for an increase or decrease in trends in the frequency of the behavior in question according to the Mann–Kendall test based on the Kendall’s tau. The numbers in the pool vary depending on the respondent’s ability to observe the behavior.

Behavior	Cat’s Age in Years: Proportion (%)			
	**<1**	**1–2**	**3–9**	**>9**
Eating weekly	71/76 (93.4%)	352/469 (75.1%)	627/1002 (62.6%)	325/565 (57.5%)
Appearing ill before eating	0/68 (0%)	17/449 (3.8%)	53/937 (5.7%)	50/531 (9.4%)
Vomiting after eating	2/73 (2.7%)	85/429 (19.8%)	336/921 (36.5%)	298/522 (57.1%)
Mostly grass eaten	21/68 (30.9%)	211/402 (52.5%)	444/845 (52.5%)	305/486 (62.8%)

**Table 2:** Cats’ frequency of eating plants and behaviors prior to and following eating plants, from Survey 2. In several instances, the behaviors before or after eating plants were not clear to the owner, so these responses were not included in the percentages.

Behavior	Proportion of Cats (%)
Eating plants at least 6 times	725/1021 (71%)
Eating plants more than 10 times Never seen eating plants	622/1021 (61%) 112/1021 (11%)
Appearing ill before eating	92/1021 (9%)
Vomiting after eating	276/1021 (27%)

**Table 3:** Frequency of eating plants and behaviors prior to and following eating plants in short-haired and long-haired cats from Survey 2. Cats designated as domestic short hair are compared with domestic long hair and Burmese with Siamese.

Behavior	Number of Cats (%)			
	Domestic Short	Domestic Long	Burmese	Siamese
Total N	373	64	17	23
Eating plants more than 10 times	310 (83%)	54 (85%)	8 (47%)	12 (52%)
Frequency:				
Once per day	101 (27%)	18 (28%)	3 (18%)	4 (17%)
Once per week	142 (38%)	24 (38%)	4 (24%)	5 (22%)
Appearing ill before eating plants	34 (9%)	6 (9%)	6 (36%)	5 (22%)
Vomiting after eating plants	97(26%)	12 (19%)	9 (50%)	11 (47%)

## Data Availability

The surveys and data presented in this study are openly available in Figshare at 10.6084/m9.figshare.12831002, accessed on 21 June 2021.

## Supplementary Materials

The following are available online at https://www.mdpi.com/article/10.3390/ani11071853/s1, File S1: Survey 1, File S2: Survey 2
