# Supplementary material for: Characteristics of Plant Eating in Domestic Cats
Source: Animals (Basel). 2021 Jun 22;11(7):1853. doi: 10.3390/ani11071853 (PMC8300339; doi:10.3390/ani11071853)
Supplement: Supplementary file 1 [file animals-11-01853-s001.zip › SUP/Supplementary File 1.pdf]

animals MDPI

Supplementary File 1 - Survey 1

Cats and Plants

********* A scientific study of grass and plant-eating behavior in cats ********* Companion Animal Behavior Program, UC Davis School of Veterinary Medicine

Why do cats eat grass or other types of plants? This survey, conducted by the UC Davis School of Veterinary Medicine, is the first to scientifically investigate this question. Your participation in this study is VERY important. Please answer as completely and as honestly as possible. ONLY TAKE THE SURVEY ONCE, otherwise our results will not be accurate.

This survey should take approximately 15-20 minutes. When the study is completed, the results will be published and made available to the public. Thank you again for your participation!

1. Household Information

Please read the questions and follow the instructions CAREFULLY, otherwise we will be unable to use your answers.

1.1. NUMBER OF CATS: Total number of cats in your household:

1.2. CATS THAT EAT/CHEW PLANTS: To your knowledge, how many of your CATS have eaten or chewed plants at least once?

2. Cat Information

NOTE For the remainder of the survey, please answer the following questions for the ONE CAT in your household that eats or chews plants MOST frequently. If you have only one cat in your household, answer the following questions for that cat. If your cat DOES NOT chew or eat plants, please press the "Submit" button above; you do not need to complete the survey.

2.1. BREED QUESTION: What breed is your cat (choose one)?

I Domestic Short-haired (DSH) - MIXED BREED cat with short-hair

I Domestic Medium-haired (DMH) - MIXED BREED cat with medium length

I hair

I Domestic Long-haired (DLH) - MIXED BREED cat with long-hair

I Abyssinian

I American Bobtail

I American Curl

I American Shorthair I American
Wirehair I Balinese

I Birman

I Bombay

I British Shorthair

I Burmese

I Chartreux

I Colorpoint Shorthair

I Cornish Rex

I Devon Rex

I Egyptian Mau

I European Burmese

I Exotic

I Havana Brown

I Himalayan

I Japanese Bobtail

I Javanese
I Korat I
LaPerm

I Maine Coon

I Manx

I Norwegian Forest Cat

I Ocicat I
Oriental I
Persian

I Ragamuffin

I Ragdoll

I Russian Blue
I Scottish Fold
I Selkirk Rex I
Siamese

I Siberian I
Singapura I
Somali I
Sphynx I
Tonkinese

I Turkish Angora
I Turkish Van I
I do not know

I Other, Please Specify:

2.2. GENDER QUESTION: What is the sex of your cat?

2.3. AGE QUESTION: How old is your cat (approximate age)?

2.4. DURATION IN HOUSEHOLD: How long has this cat been in your household?

2.5. HOUSING QUESTION: Is your cat (choose one):

I Indoor only (Not purposely let outside)

I Primarily indoor/Supervised outdoor (Spends some supervised time outdoors)

I Primarily indoor/Unsupervised outdoor (Spends some unsupervised time outdoors)

I Indoor/Outdoor (Spends about equal amounts of time indoors and unsupervised outdoors)

I Primarily outdoor (Usually outdoors but allowed to come inside)

animals MDPI

I Outdoor only (Outdoor except for rare circumstances, for example, when sick)

I Other, Please Specify:

2.6. REASON FOR ACQUISITION: Is your cat primarily a (choose one):

I Family member/Companion

I Show/Competition Cat

I Breeder - Breeding for show/competi-
tion I Breeder - Breeding for companion/pet
I Barn Cat - Pest control/Hunter

I Neighborhood cat/Stray

I Other, Please Specify:

2.7. CARETAKER QUESTION: Are you the primary caretaker of this cat?

I Yes

I No

2.8. TIME SPENT WITH YOUR CAT: On average, how many hours per day do YOU spend with your cat (able to observe his/her behavior)?

3. Plant Eating Behavior

NOTE Please continue to answer the following questions for the ONE cat in your household that eats or chews plants MOST frequently (same cat as in section 2). If you have only one cat in your household, answer the following questions for that cat.

3.1. PLANT EATING/CHEWING QUESTION: Do you think your cat eats or chews plants?

I Yes

I No

3.2. DIRECT OBSERVATION OF PLANT EATING/CHEWING: Have you SEEN your cat eating or chewing plants at least once?

I Yes

I No

3.3. OTHER EVIDENCE OF PLANT EATING/CHEWING: Other than witnessing your cat, do you have other evidence that he/she eats or chews plants?

I Yes

I No

3.4. TYPES OF EVIDENCE: If you answered "Yes" to question 3.3 above, what type(s) of evidence do you have? (Choose all that apply) If you answered "No", please check "Not applicable".

I Found plant material in his/her feces

I Found plant material in his/her vomit

I Found plant material in his/her mouth or teeth

I Other people have told me

I Found chewed plant material in your home or yard

I Had medical problems from eating/chewing plants (For example - blocked intestine, poisoning, etc.)

I Other, Please Specify:

I Not applicable

3.5. TOTAL NUMBER OF TIMES PLANT EATING/CHEWING HAS BEEN OBSERVED: Approximately how many times have you SEEN your cat eat or chew plants throughout his/her life?

3.6. AGE OF YOUR CAT WHEN PLANT EATING/CHEWING STARTED: At what age did you first notice your cat eating or chewing plants?

3.7. FREQUENCY OF PLANT EATING/CHEWING: About how frequently does your cat eat or chew plants?

3.8. EATING VS. CHEWING PLANTS: Under MOST circumstances, does your cat (choose one):

I Chew and swallow plant material (eats it) at least sometimes

I Chew but doesn't seem to swallow plant material (chews it only)

I Lick but doesn't chew plant material

I Eat or chew plant material while eating other objects (For example - treats, dirt, material, etc.)

I Eat or chew plant material incidentally while playing (For example - while playing with objects in the yard)

I Eat or chew plant material incidentally while grooming

I I do not know

I Other, Please Specify:

3.9. ACCESS TO PLANTS: Where does your cat have ACCESS to plants? (Choose all that apply)

I Inside your home (For example - houseplants, floral arrangements, etc.)

I Inside your home - plants accidentally tracked inside (For example - leaves, grass, etc.)

I Inside your home - groceries (vegetables, fruits or other plants)

I On your deck or patio

I In your yard - supervised (you are outside watching your cat)

I In your yard - unsupervised

I Outdoors/Neighborhood - supervised (For example - on leashed walks or outings)

I Outdoors/Neighborhood - unsupervised

I Intentionally offered to him/her as a dietary supple-
ment I Intentionally offered to him/her as a toy for enrichment
I Other, Please Specify:

3.10. LOCATION(S) WHERE PLANT EATING/CHEWING HAS BEEN OBSERVED: Where have you SEEN your cat eat or chew plants? (Choose all that apply)

I Inside your home (For example - houseplants, floral arrangements, etc.)

I Inside your home - plants accidentally tracked inside (For example - leaves, grass, etc.)

I Inside your home - groceries (vegetables, fruits or other plants)

I On your deck or patio

I In your yard - supervised (you are outside watching your cat)

I In your yard - unsupervised (spying; cat unaware that you are watching)

I Outside/Neighborhood - supervised (For example - on leashed walks or outings)

I Outside/Neighborhood - unsupervised

I Intentionally offered to him/her as a dietary supplement I Intentionally offered to him/her as a toy for enrichment I Other, Please Specify:

3.11. TYPES OF PLANTS YOU HAVE OBSERVED YOUR CAT EATING/CHEWING: What types of plants have you SEEN your cat eat or chew? (Choose all that apply)

I Grass - long blades (For example - weeds)

I Grass - short blades (For example - lawn grass)

I Grass - clippings

I Grass - sold commercially (Cat Grass Plus, OzzyAtt Cat Grass Kit, etc.)

I Catnip - Fresh I Catnip - Dried I Green leaves

I Brown/dried leaves

I Flowers or petals

I Berries, fruit or vegetables

I Green plant stems

I Brown/dried plant stems

I Sticks, twigs or bark

I Roots

I Other, Please Specify:

I Not Applicable

3.12. TYPES OF PLANTS YOU SUSPECT YOUR CAT OF EATING/CHEWING: Excluding those plants that you have witnessed your cat eating or chewing, what OTHER types of plants do you SUSPECT your cat eats or chews based on indirect evidence (for

example, seeing plant material in his/her feces, finding chewed plants, etc.)? (Choose all that apply)

I Grass - long blades (For example - weeds)

I Grass - short blades (For example - lawn grass)

I Grass - clippings

I Grass - sold commercially (For example - Cat Grass Plus, OzzyAtt Cat

I Grass Kit,
etc.) I Catnip - fresh
I Catnip -
dried I Green leaves

I Brown/dried leaves

I Flowers or petals

I Berries, fruit or vegetables

I Green plant stems

I Brown/dried plant stems

I Sticks, twigs or bark

I Roots

I Other, Please Specify:

I Not Applicable

3.13. TYPES OF PLANTS MOST FREQUENTLY EATEN/CHEWED: Based on either direct observation or other evidence, what type of plant does your cat MOST FREQUENTLY eat or chew? (Choose ONE answer)

I Grass - long blades (For example - weeds)

I Grass - short blades (For example - lawn grass)

I Grass - clippings

I Grass - sold commercially (For example - Cat Grass Plus, OzzyAtt Cat

I Grass Kit,
etc.) I Catnip - fresh
I Catnip -
dried I Green leaves

I Brown/dried leaves

I Flowers or petals

I Berries, fruit or vegetables

I Green plant stems

I Brown/dried plant stems

I Sticks, twigs or bark

I Roots

I Other, Please Specify:

I Not Applicable

3.14. NAME OF PLANT EATEN OR CHEWED MOST FREQUENTLY: If you know the SPECIFIC type and part of the plant your cat most frequently eats or chews, please write it below. (For example - Oat grass, commercially produced, eats tips only; Chinese peas - eats pea pods only.).

If you do not know the specific type of plant, please check "I do not know".

animals

I I do not know

3.15. BEHAVIOR(S) INVOLVED IN EATING/CHEWING PLANTS: Does your cat exhibit any particular patterns to his/her plant eating/chewing behavior? (Choose all that apply)

I I have not observed him/her - I do not know

I No particular pattern observed

I Eats/chews plants when apparently feeling "ill" (For example - inactive, not eating, hiding, etc.)

I Eats/chews plants in specific locations (For example - friend's home, specific corner of yard, etc.)

I Eats/chew splants if other pets are also eating plants

I Would eat/chew plants all the time if I let him/her

animals MDPI

I Eats/chews plants following specific events (For example - after being brushed, after coming home from the vet, etc.)

I Eats/chews plants at certain times of the day (For example - morning, evening, etc.)

I Eats/chews plants at certain times of the year (For example - spring, fall, etc.)

I Eats/chews plants when "bored"

I Eats/chews plants after certain weather conditions (For example – after it has rained)

I Eats/chews plants that other animals have urinated on

I Other, Please Specify:

I None of the above

3.16. CAT'S BEHAVIOR BEFORE EATING/CHEWING PLANTS: With respect to the type of plant your cat MOST COMMONLY eats or chews (Question 3.13) - Select the behavior your cat MOST COMMONLY engages in during the hour BEFORE your cat eats THIS SPECIFIC TYPE OF PLANT. (Choose one)

I Unable to observe - I do not know

I Goes about his/her normal life; acts normally which may include sleeping, eating, drinking, grooming, etc.

I Vomits AND shows signs of feeling "ill" (For example - depression, loss of appetite, inactivity, etc.)

I Does NOT vomit, but shows signs of feeling "ill" (For example - depression, loss of appetite, inactivity, etc.)

I Vomits, but does NOT appear "ill"

I Other, Please Specify:

3.17. CAT'S BEHAVIOR AFTER EATING/CHEWING PLANTS: With respect to the type of plant your cat MOST COMMONLY eats or chews (Question 3.13) - Select the behavior your cat MOST COMMONLY engages in during the hour AFTER your cat eats THIS SPECIFIC TYPE OF PLANT. (Choose one)

I Unable to observe - I do not know

I Goes about his/her normal life; acts normally which may include sleeping, eating, drinking, grooming, etc.

I Vomits AND shows signs of feeling "ill" (For example - depression, loss of appetite, inactivity, etc.)

animals

I Does NOT vomit, but shows signs of feeling "ill" (For example - depression, loss of appetite, inactivity, etc.)

I Vomits, but does NOT appear "ill"

I Other, Please Specify:

3.18. FREQUENCY OF VOMITING BEFORE EATING/CHEWING PLANTS: How frequently does your cat vomit BEFORE eating or chewing plants? (Choose one answer)

I Never vomits before eating or chewing plants (0% of the time)

I Rarely vomits before eating or chewing plants (Less than 10% of the time) I Occasionally vomits before eating or chewing plants (10-25% of the time) I Sometimes vomits before eating or chewing plants (205-50% of the time)
I Often vomits before eating or chewing plants (50-75% of the time)

I Frequently vomits before eating or chewing plants (75-90% of the time)

I Typically vomits before eating or chewing plants (Greater than 90% of the time)

I Always vomits before eating or chewing plants (100% of the time)

3.19. FREQUENCY OF VOMITING AFTER EATING/CHEWING PLANTS: How frequently does your cat vomit AFTER eating or chewing plants? (Choose one answer)

I Never vomits after eating or chewing plants (0% of the time)

I Rarely vomits after eating or chewing plants (Less than 10% of the time) I Occasionally vomits after eating or chewing plants (10-25% of the time) I Sometimes vomits after eating or chewing plants (25-50% of the time)
I Often vomits after eating or chewing plants (50-75% of the time)

I Frequently vomits after eating or chewing plants (75-90% of the time)

I Typically vomits after eating or chewing plants (Greater than 90% of the time)

I Always vomits after eating or chewing plants (100% of the time)

3.20. TIME INTERVAL BETWEEN VOMITING AND EATING/CHEWING PLANTS: If your cat vomits BEFORE eating or chewing plants – On average, how many hours after vomiting does your cat eat or chew plants?

I Not Applicable

3.21. TIME INTERVAL BETWEEN EATING/CHEWING PLANTS AND VOMITING: If your cat vomits AFTER eating or chewing plants – On average, how many hours after eating or chewing plants does your cat vomit?

I Not Applicable

3.22. ASSOCIATION BETWEEN VOMITING AND EATING/CHEWING CERTAIN TYPES OF PLANTS: Does your cat regularly vomit after eating certain types of plants? (Choose all that apply)

I Grass (long blades, short blades, clippings, commercially grown)

I Catnip (fresh or dried)
I Leaves (green or dried) IFlowers or petals

I Berries, fruits or vegetables I
Plant stems (green or dried) I
Sticks, twigs or bark

I Roots

I Other, Please Specify:

I Not Applicable

3.23. PLANTS IN FECES: Have you ever seen grass or other plant material in your cat's FRESH feces/droppings?

I Yes

I No

4. Diet

4.1. DIET QUESTION: Select all types of food your feed your cat more than once per week. (Choose all that apply)

I Moist food/canned or foil packaged

I Dry food/kibble

I Semi-moist food (For example - Purina Happy Cat moist, etc.)

I Home-cooked diet

I Raw food diet (For example - chicken back/necks, frozen commercial raw food diet, etc.)

I Commercial treats - Dry (For example - Pounce Hairball Crunchy Treat, etc.)

I Commercial treats - Semi-moist (For example - Pounce moist treats, Friskies cat treats, etc.)

I Commercial treats - Dried shrimp or fish flakes

I Treats - "People food"/Left-overs

4.2. PRIMARY DIET QUESTION: Your cat's diet consists primarily (>50%) of (Choose one answer):

I Moist food/canned or foil packaged

I Dry food/kibble

I Semi-moist food (For example - Purina Happy Cat moist, etc.)

I Home-cooked diet

I Raw food diet (For example - chicken back/necks, frozen commercial raw food diet, etc.)

I Commercial treats - Dry (For example - Pounce Hairball Crunchy Treat, etc.)

I Commercial treats - Semi-moist (For example - Pounce moist treats, Friskies cat treats, etc.)

I Commercial treats - Dried shrimp or fish flakes

I Treats - "People food"/Left-overs

I Other, Please Specify:

4.3. TYPE OF FOOD: This food is (choose one):

I Commercial - Premium Label (For example - Eukanuba, Science Diet, Purina, Alpo, etc.)

I Commercial - Private Label (For example - Costco brand, Safeway brand, Old Mother Hubbard, etc.)

I Prescription - Only available through your veterinarian (For example - Hill's w/d, IVD duck and potato, Purina HA, etc.)

I Home-cooked - Veterinarian recommended (For example - home-cooked diet for food allergies or intestinal disease, etc.)

I Home-cooked diet - Not specifically recommended by a veterinarian I Raw food diet - Commercial (For example - Steve's Real Food, Primal I Pet Food, Animal Food Services Pet Patties, Sojourner Farms, etc.)

I Raw food diet - Home prepared (For example - chicken necks/backs, etc.)

I Other, Please Specify:

4.4. DURATION OF CURRENT DIET: How long has your cat been on this same diet?

animals

4.5. DIET MODIFICATION: Did you change your cat's diet after noticing that he/she ate or chewed plants? (Choose all that apply)

I I did NOT change my cat's diet after noticing he/she ate or chewed plants

I Changed type or brand of food - Specifically to increase fiber

I Changed type or brand of food - For other reasons (For example - to try a prescription diet, to try something more tasty, etc.)

I Added vitamin or mineral supplement to diet (For example - Barley Cat, vitamin C, MSM, etc.)

I Added vegetables or other plant material to diet (For example – started feeding vegetables, commercial grass, etc.)

I Other, Please Specify:

4.6. AFFECT OF DIET MODIFICATION ON PLANT EATING/CHEWING: If you changed your cat's diet or added supplements, did it affect the frequency of plant eating or chewing? If you did not change your cat's diet, please mark "Not Applicable". (Choose one answer)

I No change

I Increased the frequency of plant eating/chewing I Decreased the frequency of plant eating/chewing I Other, Please Specify:

I Not Applicable

5. Medical History

5.1. CURRENT MEDICAL HISTORY: Does your cat CURRENTLY have any of the following diseases? (Choose all that apply)

I Not currently ill - Does not currently have any disease

I Stomach or Intestine/GI (For example - vomiting, diarrhea, IBD, intestinal worms, etc.)

I Skin/Dermatologic (For example - allergies, fleas, hair loss, etc.)

I Urinary (For example - renal failure, urinary tract infection, cystitis, bladder or kidney stones, etc.)

animals

I Heart/Cardiac (For example - heart failure, thickened heart, HCM, abnormal heart beats or rhythm, etc.)

I Lungs/Respiratory (For example - asthma, etc.)

I Endocrine (For example - hyperthyroid, diabetes, etc.)

I Eyes/Ophthalmologic (For example - ulcers, runny eyes, etc.,)

I Musculoskeletal (For example - arthritis, fractures, etc.)

I Neurologic (For example - seizures, back or neck pain or trauma, etc.)

I Cancer

I Other, Please Specify:

5.2. WORMS IN FECES: Have you or your veterinarian ever seen worms (tapeworms, roundworms, hookworms, whipworms, etc.) or worm eggs in your cat's feces/droppings?

I Yes

I No

I I do not know

5.3. PARASITE CONTROL: Does your cat regularly receive any of the following medications? (Choose all that apply)

I Not receiving any medications regularly

I I do not know

I Flea/tick control - Topical (on the skin) (For example - Frontline, Advantage, etc.)

I Flea/tick control - Pills (For example - Program, Capstar, etc.)

I Flea/tick control - Injectable (For example - Program injectable, etc.)

I Flea/tick control - Collar (For example - Preventic, Hartz, etc.)

I Flea/intestinal worms/heartworm/mites - Topical (on the skin) (For example - Revolution, etc.)

I Heartworm Only - Pill or Chew (For example - Heartgard, etc.)

I Heartworm/Intestinal Parasites - Pill (For example - Interceptor)

I Herbal or other supplements to treat fleas/ticks (For example - garlic, etc.)

I Herbal or other supplemetnts to treat intestinal parasites or worms (For example - mineral oil, etc.)

I Other, Please Specify:

5.4. CURRENT MEDICATIONS: Is your cat CURRENTLY receiving or has received in the past MONTH any of the following medications? (Choose all that apply)

I Not currently receiving any medications; has not received any medications in the past month

I I do not know

I Deworming medication (For example - Drontal, Droncit, Panacur,

I D-worm, Strongid, etc.)

I Steroids (For example - prednisone or prednisolone)

I Anti-seizure medication (For example - phenobarbitol or potassium bromide)

I Appetite stimulants (For example - cyproheptidine)

I Thyroid medication (For example - Tapazol or other medication for hyperthyroidism)

I Pain medication (For example - aspirin, Rimadyl, Metacam. etc.)

animals

- I Behavior drugs (For example - Elavil/amitriptyline, Prozac/fluoxetine, Buspar, Valium, Xanax, etc.)
- I Antacids (For example - Pepcid, Zantac, Prilosec,Tagamet, etc.)
- I Eye medication with steroids (For example - Trioptic, Vetropolcin, Neo-Predf, BNP with hydrocortisone, etc.)
- I Ear medication with steroids (For example - Synotic, Tresaderm, Panalog, Gentocin Otic, etc.)
- I Antibiotics (For example - Clavamox, Amoxi-drops, Baytril, cephalexin, etc.)
- I Sedatives or tranquilizers (For example - acepromazine, etc.)
- I Other, Please Specify:
- I Not currently receiving any medications

This survey was created using the Survey-Suite Survey Generation Tool
