# Supplementary material for: Characteristics of Plant Eating in Domestic Cats
Source: Animals (Basel). 2021 Jun 22;11(7):1853. doi: 10.3390/ani11071853 (PMC8300339; doi:10.3390/ani11071853)
Supplement: Supplementary file 1 [file animals-11-01853-s001.zip › SUP/Supplementary File 2.pdf]

animals MDPI

Supplementary File 2 - Survey 2

2016 Life with Your Cat Understanding Feline Behaviors

1. What is the sex of the specified cat?
   I Male neutered
   I Male intact (not neutered)
   I Female spayed
   I Female intact (not spayed)

2. What is the age of the specified cat?
   I Less than 1 year
   I 1-3 years
   I 4-6 years
   I 7-10 years
   I 11-15 years
   I 16-20 years
   I Over 20 years
   I Too unsure to indicate any of the above

3. Please indicate whether the specified cat is totally indoors, totally outdoors or indoor-outdoor.
   I Indoor cat only
   I Outdoor cat only
   I Indoor-outdoor cat; allowed outdoors

4. This question deals with how long have you known the specified cat.
   I Less than 1 year
   I 1-3 years
   I 4-6 years
   I 7-10 years
   I Over 10 years

5. In the time you have known the specified cat, approximately how many hours per day, on average, have you been in a position to notice the cat's behavior such as yawning, purring and eating plants?
   I Less than 1 hour per day
   I 1-2 hours per day I
   3-6 hours per day I
   Over 6 hours per day

6. What was the source of the specified cat?
   I Purebred breeder
   I From friend, neighbor or other person who had a litter of kittens
   I Animal shelter or humane society

- I A feral cat that I took in
- I Other (please specify)

animals

7. What is the breed or breed type of this cat?
   I Domestic shorthair
   I Domestic longhair I
   Abyssinian
   I Bengal
   I Burmese I
   Cornish Rex I
   Exotic
   I Norwegian Forest
   I Maine Coon
   I Manx I
   Oriental I
   Persian I
   Ragdoll
   I Russian Blue
   I Siamese I
   Sphynx I
   Tonkinese
   I I do not know
   I Other (please specify)

8. Number of cats in your household
   I One
   I Two
   I Three
   I Four
   I More than four

9. Plant eating. It is known that some cats eat grass or other plants. This question deals with whether or not your specified cat has access to plants. Please state where, if at all, your cat has access to plants. You may indicate more than one area of access to plants.
   I Plant eating. It is known that some cats eat grass or other plants. This question deals with whether or not your specified cat has access to plants. Please state where, if at all, your cat has access to plants. You may indicate more than one area of access to plants.
   I Outdoors on a leash
   I Outdoor fenced area with plants
   I Plants that I have indoors, but not plants intentionally provided for the cat
   I A bed of grass indoors that I provide for the cat on a regular basis
   I The cat is indoors only with no plants available
   I Other (please specify)

10. If you provide a bed of grass, share with us the reasons you provide this indoor grass. Choose all that apply.

animals

I I do not provide a bed of grass

animals

MDPI

I The cat enjoys eating the grass
I I think the grass is good for the cat
I The cat knows she/he needs the fiber
I This keeps my cat from eating house plants I My veterinarian recommended the grass I I want the house to seem more natural
I Other (please specify)

11. How many times, total, have you seen your cat eating grass or other plants.
I More than 10 times
I At least 6 times but less than 10
I More than once but less than 6 times
I Just one time
I I have never seen my cat eating plants

12. What is your best estimate of the frequency with which your cat engages in eating grass or other plants?
I 1 time per day or more
I Less than once a day, but at least 1 time per week
I Less than once a week, but at least 1 time per month
I Less than once a month, but at least 1 time per year
I Less than once a year
I This question does not apply because, as far as I know, my cat does not eat plants

13. Please answer this question about whether or not your cat regularly appears sick or ill prior to eating grass or other plants.
I Plant eating is so infrequent that I cannot answer this question
I The cat seems to be sick only occasionally before eating plants
I The cat seems to be frequently sick before eating plants
I The cat seems to be almost always normal before eating plants

14. Please answer this question about whether or not your cat regularly vomits within an hour after eating grass or other plants.
I Plant eating is so infrequent that I cannot answer this question
I The cat vomits only occasionally after eating plants
I The cat frequently vomits after eating plants
I The cat seems to be almost always normal after eating plants

15. Response to catnip. Have you in the past, or do you currently, provide some sort of catnip to your cat in the way of a stuffed toy (mouse), fresh catnip leaves or dried leaves?
I I have not provided a catnip source of any type
I I provide a catnip source on a weekly basis

animals

I I provide a catnip source on a monthly basis
I I provide a catnip source more than once a year but less than monthly

16. Catnip source. What is the type of catnip most frequently provided? You may choose more than one response.
    I Stuffed catnip toy such as mouse
    I Fresh catnip leaves
    I Dried catnip leaves
    I Catnip material sprayed on objects I
    I have not provided catnip of any type I
    Other (please specify)

17. Response to catnip. If you have provided catnip for this cat at least once, please choose the response below that best describes the cat's behavior, after you have provided catnip.
    I Does not respond to catnip and never did
    I Responds to catnip just a small percentage of times
    I Responds to catnip most of the time
    I Responds to catnip virtually every time I offer it
    I This question does not apply because I have not provided catnip of any type
    I Other (please specify)

18. Response to catnip. What response below best describes your cat's reaction to catnip. Choose all that apply.
    I Plays with the catnip like kittens play
    I Bites into the catnip source
    I Licks the catnip
    I Rolls or rubs on the catnip source
    I Eats the catnip
    I My cat does not respond to catnip as far as I know
    I This question does not apply because I have not provided catnip of any type
    I Other (please specify)

19. Please indicate the number of times, over the last year, that YOUR CAT had an illness as described above.
    I My cat had no such illnesses
    I 1 time
    I 2 times
    I 3 times
    I More than 3 times
    I I do not know
